# Secure and Efficient Lattice-Based Ring Signcryption Scheme for BCCL

**DOI:** 10.3390/e27101060

**Published:** 2025-10-12

**Authors:** Yang Zhang, Pengxiao Duan, Chaoyang Li, Haseeb Ahmad, Hua Zhang

**Affiliations:** 1 College of Software Engineering, Zhengzhou University of Light Industry, Zhengzhou 450001, China; zhangyang@zzuli.edu.cn (Y.Z.); 2009046@zzuli.edu.cn (H.Z.); 2 Department of Computer Science, National Textile University, Faisalabad 37610, Pakistan; haseeb_ad@ntu.edu.pk

**Keywords:** blockchain, cold chain logistics, ring signcryption, lattice

## Abstract

Blockchain-based cold chain logistics (BCCL) systems establish a new logistics data-sharing mechanism with blockchain technology, which destroys the traditional data island problem and promotes cross-institutional data interoperability. However, security vulnerabilities, risks of data loss, exposure of private information, and particularly the emergence of quantum-based attacks pose heightened threats to the existing BCCL framework. This paper first introduces a transaction privacy preserving (TPP) model for BCCLS that aggregates the blockchain and ring signcryption scheme together to strengthen the security of the data exchange process. Then, a lattice-based ring signcryption (LRSC) scheme is proposed. This LRSC utilizes the lattice assumption to enhance resistance against quantum attacks while employing ring mechanisms to safeguard the anonymity and privacy of the actual signer. It also executes signature and encryption algorithms simultaneously to improve algorithm execution efficiency. Moreover, the formal security proof results show that this LRSC can capture the signer’s confidentiality and unforgeability. Experimental findings indicate that the LRSC scheme achieves higher efficiency compared with comparable approaches. The proposed TPP model and LRSC scheme effectively facilitate cross-institutional logistics data exchange and enhance the utilization of logistics information via the BCCL system.

## 1. Introduction

Blockchain-based cold chain logistics (BCCL) is a new logistics platform that establishes a distributed data-sharing mechanism among different cold chain logistics institutions [1]. It promotes logistics data utilization, thereby enhancing the delivery efficiency of products within the cold chain. Cold chain logistics data typically features multimodality, including structured text information (such as orders and temperature records), semi-structured or unstructured image data (such as photos of packaging and cargo status), and video data (such as transportation monitoring videos), which contain a large amount of sensitive information and commercial privacy. However, security concerns, including risks of data loss, exposure of private information, and threats from quantum-based attacks, increase with the number of terminal devices [2]. Therefore, seeking more efficient and anti-quantum encryption methods is more essential for safeguarding privacy in logistics data-sharing processes within BCCL systems.

Blockchain technology changes the data island problem in traditional logistics systems and helps to achieve data sharing and interoperability across different cold chain logistics institutions [3]. Especially for cold chain products, where it is important to pay special attention to time and safety, it is urgent to make good use of logistics data and advance the innovation and growth of cold chain logistics. In recent years, some blockchain-based frameworks have been proposed, and these proposals focus on the system architecture with consortium [4,5,6,7,8], private [9,10], or hybrid [11,12,13] blockchain technologies. Meanwhile, some other proposals focus on security issues and introduce new BCCL frameworks with video encryption [14,15], encryption/decryption [16,17], signature [18,19], signcryption [20,21,22], secret sharing [23,24], and key agreement [25] algorithms. These cryptographic algorithms protect logistics data and user privacy, utilizing difficult mathematical problems to construct a defense line for network security. However, the swift advancement of quantum technologies poses a significant risk to the current BCCL system with classical cryptographic algorithms [26]. Most cryptographic algorithms founded on large integer factorization and discrete logarithm cannot effectively resist quantum attacks from Shor and Grover algorithms [27,28].

Post-quantum cryptography (PQC) offers algorithms that provide stronger resistance against quantum attacks, which generally include lattice-based, code-based, hash-based, and multivariate cryptography [29]. Code-based cryptography is constructed with coding theory and error-correcting code technologies, but it is not suitable for creating an encryption scheme with a large public key [30]. Hash-based cryptography is constructed by a random hash function with sufficient length, which generally has a large key size and computational complexity [31]. Multivariate cryptography can be used to create a more efficient signature scheme, but it is not practical with a large public key [32]. Although lattice-based cryptography has a slightly larger key size, it has high security, simple implementation, and efficient performance [33]. Now, it is the first choice of the PQC standard and receives extensive research. In current information systems, the encryption and decryption operations of the signature are performed at the same time, and the signcryption algorithm appears. This cryptographic primitive was first introduced in 1997 [34], which greatly improved the efficiency of secure information processing. It generally contains three kinds of algorithms: PKI-based, identity-based, and certificateless. There are also some lattice-based signcryption schemes have been proposed in recent years [35,36,37,38,39,40,41,42]. However, existing schemes still face challenges in both security and efficiency. Some approaches focus exclusively on confidentiality and unforgeability, without providing anonymity protection for the signcrypter, which limits their applicability in privacy-sensitive scenarios. Others incorporate anonymity mechanisms but either omit ring structures or introduce high computational complexity, resulting in significant overhead during the signcryption and unsigncryption phases. Such inefficiencies reduce their suitability for real-time communication and lightweight applications. In contrast, the proposed lattice-based ring signcryption (LRSC) scheme simultaneously ensures confidentiality, unforgeability, and signer anonymity while maintaining practical efficiency, thereby addressing the shortcomings of existing research. Moreover, algorithms intended for the BCCL system must be specifically tailored to meet its requirements.

To satisfy the security concerns in the BCCL system, a TPP model and LRSC scheme have been proposed. This paper has three main contributions.

1. A TPP model for the BCCL system has been introduced with blockchain and ring signcryption technologies. The blockchain serves as the data-sharing bridge among different cold chain logistics institutions. The ring signcryption serves as the key security mechanism ensuring the protection of logistics information and user privacy during cross-institutional data exchanges within the BCCL system.
2. A LRSC scheme has been proposed with lattice theory and a ring mechanism. Lattice theory enables anti-quantum security for the BCCL system, and the ring mechanism protects the signer’s privacy. Meanwhile, the signcryption mechanism effectively saves the algorithm’s execution efficiency.
3. Security demonstrations for confidentiality and unforgeability have been established within the framework of the random oracle model. The proposed LRSC scheme captures these two security properties. Meanwhile, the experiment is executed, and the findings demonstrate the efficiency of both the TPP model and the LRSC scheme.

Next, Section 2 presents the related work, Section 3 introduces a TPP model, Section 4 proposes the LRSC scheme, Section 5 shows the formal security proof, Section 6 presents the performance simulation, and Section 7 concludes this work.

## 2. Related Work

This section begins with an overview of the advancements in BCCL framework research, then it presents the privacy-preserving methods for the BCCL. In addition, post-quantum privacy-preserving methods are reviewed.

### 2.1. BCCL Frameworks

Blockchain technology has been employed to create a distributed logistics data-sharing platform, addressing the data island issue in conventional cold chain logistics systems. With different kinds of blockchain technologies, some new BCCL frameworks have been introduced. The consortium blockchain is more efficient than public chains and is suitable for cooperation between specific industries or institutions. Song et al. [4] employed cyber-physical systems in combination with blockchain technology to form a consortium for the agricultural supply chain, and they introduced a consensus protocol to achieve transaction consistency. He et al. [5] introduced a logistics system built on consortium blockchain technology to enhance traceability in logistics operations, and they also designed a zero-knowledge proof-based scheme to strengthen data trustworthiness. Allenbrand [6] proposed a blockchain-based smart contract system to align incentives in supply chains, improving forecast coordination and reducing the bullwhip effect through sustained, reward-driven cooperation. Zhang et al. [7] introduced a blockchain-based cold-chain logistics system driven by the IoV and incorporating a linkable ring signature mechanism, ensuring secure, tamper-proof, and efficient real-time data sharing, traceability, and privacy protection. Xiong et al. [8] developed a blockchain-enabled SCN data-sharing framework integrating privacy protection, access control, and incentive mechanisms to facilitate secure, efficient, and equitable cross-domain cooperation among supply chain enterprises. The private blockchain is controlled by a single organization, which has strictly restricted access rights and participants. The transaction processing speeds are usually faster than those of a public chain. Kim et al. [9] constructed a Hyperledger Fabric-based blockchain blood cold chain system to enhance real-time data transparency, ensure secure B2B blood transactions, and improve blood usage efficiency and emergency responsiveness. Zhang et al. [10] established a blockchain-enabled traceability platform for fresh agricultural cold-chain logistics, employing alliance and private blockchains to guarantee data security, operational efficiency, and trustworthy consumer product tracking. In addition, some proposals utilize the hybrid blockchain, such as the public and consortium blockchains, the consortium, and private blockchains to flexibly use the characteristics of different blockchains. Yang et al. [11] investigated the use of public and private blockchains in the construction industry via two case analyses, demonstrating system architectures, benefits, and challenges using Ethereum and Hyperledger Fabric to enhance project lifecycle processes. Si et al. [12] proposed a blockchain system with off-chain IPFS storage and access control for secure, efficient massive data sharing, improving cold chain logistics efficiency and resource allocation in agricultural supply chains. Bottoni et al. [13] introduced a Hyperledger Fabric-based blockchain architecture using smart contracts to implement Income Sharing in supply chains, enhancing productivity, innovation, and economic returns via automated, transparent income redistribution.

Existing studies have primarily focused on constructing BCCL frameworks for cross-institutional logistics data sharing. However, while the transparency of blockchain ledgers enhances traceability and verifiability, it also introduces significant privacy and security risks. Ensuring the protection of sensitive data while maintaining system transparency and credibility has thus emerged as a critical challenge in this field, which in turn motivates in-depth research on privacy-preserving mechanisms for BCCL.

### 2.2. Privacy-Preserving Mechanisms for BCCL

Cryptography algorithms are the main security guarantee for current information systems while safeguarding system data integrity and ensuring user privacy protection. Encryption/decryption technology changes the format of plaintext to hide the information content, and it has strong security as users with the correct decryption key can obtain the original message. In cold chain scenarios, the protected objects extend beyond textual data to include sensor images and real-time video, posing higher requirements on both the performance and applicability of encryption methods. For video as a representative data type, several innovative approaches have been proposed. For instance, Gao and Zhang et al. [14] introduced the “Encrypt a Story (EAS)” method, which selectively encrypts video segments containing critical information, thereby ensuring security while significantly reducing computational overhead; in addition, a discrete sinusoidal memristive Rulkov neuron map was designed to generate pseudorandom sequences, enhancing the algorithm’s resistance to attacks. Gao and Wu et al. [15] conducted a comprehensive survey of video encryption from the perspective of chaos theory, developing a mathematical model and highlighting the unique role of chaotic systems in strengthening the security of video transmission and storage. They further classified typical chaotic systems and existing encryption algorithms, providing theoretical guidance for selecting and optimizing video encryption schemes. These studies demonstrate that video encryption requires not only a balance between security and efficiency but can also benefit from the integration of advanced chaotic systems and intelligent segmentation strategies to secure heterogeneous data in cold chain environments. Moreover, in such complex supply chain scenarios, where data types are diverse and lifecycles are long, encryption methods must also ensure traceability and long-term security at the system level. Cha et al. [16] introduced a framework combining blockchain technology with key escrow encryption to ensure traceability, data security, and long-term availability in supply chains for long-lifecycle systems, enhancing global business survivability. Din et al. [17] integrated homomorphic encryption with blockchain technology to secure IoT data, ensuring privacy, integrity, and scalability, achieving encrypted transmission and real-time, tamper-proof data processing. The signature technology involves using a private key to sign a message, allowing anyone to verify the authenticity of the signature. Mouléry et al. [19] modeled beef foodsheds as spatial archipelagos, revealing how patch connectivity and geography influence short supply chains, guiding policies to enhance local beef self-sufficiency and food security in Avignon, France. Li et al. [18] introduced a blockchain-powered cold-chain logistics system featuring a lattice-based undeniable signature mechanism, ensuring secure, quantum-resistant data sharing, enhanced privacy, and efficient, tamper-proof logistics management. Signcryption technology evolves from traditional digital signatures by integrating the processes of signing and encryption into a single operation, thereby achieving both data confidentiality and authenticity simultaneously. Zhan et al. [20] proposed a multi-mode certificateless ring signcryption scheme that incorporates full anonymity, linkable anonymity, and revocable anonymity within its framework, enabling adaptability to diverse communication scenarios and addressing the limitations of existing privacy-preserving solutions restricted to single application environments. Zhou et al. [21] combined ring signcryption with consortium blockchain technology, leveraging blockchain to resolve data dispute issues in application scenarios requiring specific access control mechanisms. They also introduced a heterogeneous ring signcryption scheme to realize privacy protection and conditional traceability. Wei et al. [22] utilized a KGC to produce partial private keys for users, thus avoiding the key escrow problem, while their identity-based ring signcryption scheme further supports both traceability and non-repudiation. Secret sharing technology semantically destroys the original message, and only users who know the parameters can correctly restore the original message. Xiong et al. [23] developed a construction supply chain framework leveraging blockchain technology and introduced a secure private-key distribution protocol with key recovery, enhancing payment security and reducing delays and costs in traditional systems. Kalyani et al. [24] enhanced blockchain-based supply chain security by introducing a hybrid Whale–Butterfly optimization algorithm for optimal key generation, improving data sanitization, restoration, and privacy in secure information sharing. The key agreement technology uses cryptographic technology to directly establish a communication key between two strangers. Vangala et al. [25] introduced AgroMobiBlock, a blockchain-based authenticated key agreement protocol for precision farming IoT networks, offering secure, low-cost communication and robust protection against various attacks with proven scalability and practicality.

Existing studies mainly focus on privacy protection mechanisms in BCCL systems. However, with the growing threat of quantum attacks, the security of traditional cryptographic algorithms is increasingly challenged. PQC, with its resistance to quantum adversaries, has emerged as a key direction for ensuring secure cross-institutional data sharing, laying the foundation for subsequent research on PQC-based privacy protection methods in BCCL.

### 2.3. Post-Quantum Privacy-Preserving Methods

PQC is a network security guarantee for the future era of quantum computers. PQC-empowered signcryption technology will also be an emerging method to achieve encryption and authentication. Ali and Obaidat [35] addressed privacy and security challenges arising from the integration of IoT technologies into modern healthcare systems by proposing a lattice-based signcryption scheme for blockchain-enabled IoT healthcare. Leveraging the quantum-resistant hardness of the LWE problem, the scheme guarantees data confidentiality and authenticity while supporting user anonymity and unlinkability on the blockchain. Xu et al. [36] introduced a novel post-quantum certificateless signcryption scheme with linkability, which minimizes the risk of privacy leakage while ensuring secure transmission of medical data. Yang et al. [37] introduced an efficient lattice-based signcryption scheme combining partitioning and tag-based key encapsulation, achieving strong security under RLWE and ISIS assumptions with reduced communication and computation overhead. Yu et al. [38] presented the L-CLSS, a lattice-based certificateless signcryption scheme offering quantum-resistant, efficient simultaneous signature and encryption with provable security under LWE and SIS assumptions. Yadav [39] proposed ALRS, an anonymous, linkable ring signcryption scheme tailored for VANET-based LBS, ensuring protection of vehicle user identities and service provider data privacy with low communication costs and maintaining query linkability. Sourav et al. [40] presented LRS-SHM, a lattice-based ring signcryption scheme with regenerated keys and a (t, n) threshold method, enhancing privacy, quantum resistance, and anonymous health record management efficiently. Bai et al. [41] proposed MLCLOOSC, a quantum-resistant module-lattice-based certificateless signcryption scheme for IoMT, enhancing confidentiality, unforgeability, and efficiency with lower computational and communication costs than existing schemes. Prajapat et al. [42] employed a lattice-based ring signcryption approach to secure VANETs within an energy-efficient consortium blockchain-enabled heterogeneous 6G network architecture for IoT devices. Even under quantum threats, the scheme ensures vehicle anonymity, cloud data confidentiality, and timely message relay.

Under the lattice assumption, existing signcryption schemes provide certain post-quantum security. However, their efficiency remains limited, and most prior work has not been specifically optimized for the cross-institutional data sharing requirements of BCCL systems. As a result, these schemes face challenges in simultaneously achieving high efficiency and scalability in practical applications. In contrast, this work not only ensures post-quantum security but also takes into account the specific characteristics and performance demands of BCCL scenarios. Specifically, a transaction privacy preserving (TPP) model for BCCL systems is constructed, along with an efficient ring signcryption scheme integrating PQC, thereby achieving a balanced trade-off between security, efficiency, and applicability.

In summary, existing studies provide an important foundation for privacy protection and post-quantum research in BCCL systems, but they remain limited in terms of efficiency and practical applicability. This work extends these studies by proposing a solution that addresses both post-quantum security and real-world operational requirements, offering a new perspective for the development of secure and efficient BCCL systems.

## 3. TPP Model for BCCL

To safeguard logistics data security and user privacy, a TPP model integrating the LRSC scheme and blockchain technology is developed.

### 3.1. Details of TPP Model

**Figure 1:**
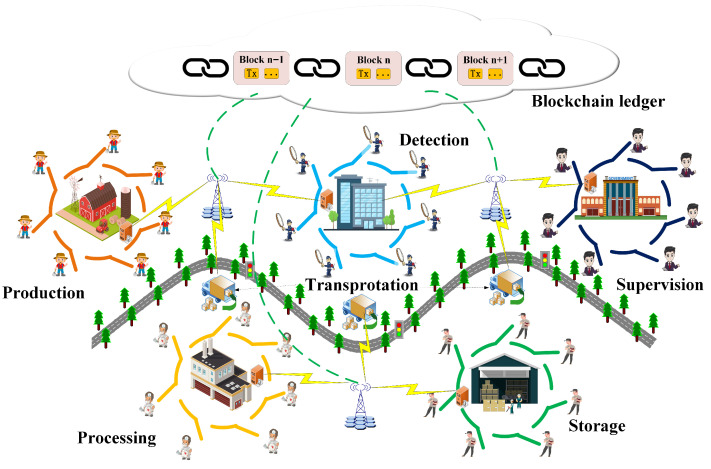
TPP model for BCCL system.

In the TPP model, the LRSC scheme performs encryption and anonymous signing within institutions in a single operation, thereby maintaining data privacy while ensuring efficient sharing, streamlining the process, and providing data integrity and resistance to quantum attacks. Within this model, blockchain functions as a platform for data exchange among cold chain logistics entities, enabling decentralized and traceable sharing. The TPP model consists of four main components: cold chain institutions, logistics vehicles, data sharing transactions, and the blockchain ledger, with a simplified structure illustrated in Figure 1.

- Cold chain institution. Different logistics institutions related to cold chain products, such as production, processing, transportation, detection, storage, and supervision, compose this BCCLS system. They serve as the blockchain nodes to maintain the BCCLS system and take responsibility for the collection, verification, and packaging of data-sharing transactions. Meanwhile, every cold chain institution is a ring. In order to improve transaction efficiency, this model utilizes the LRSC scheme to simultaneously carry out both signature and encryption processes. The ring signature mechanism can select one ring member to represent the ring and perform the signing operation. It can protect the user’s privacy while increasing signature efficiency.
- Cold chain logistics vehicle. When the cold chain products are transmitted among different cold chain institutions, the safety of the transportation process is an important part of ensuring product safety. The vehicle serves as the most important part of the BCCL system, which guarantees the transportation security of cold chain products. Temperature, humidity, route, duration, etc., during transportation are all very important data. The collection, sharing, and storage of these data provide strong evidence for cold chain product security disputes.
- Data-sharing transaction. When the cold chain products arrive at the next institution, they should be safe and qualified. Therefore, the logistics data should be shared and public for every institution and consumer. The data-sharing transaction establishes a data exchange channel with the blockchain. The related transportation data and operation records are collected and formed with transactions, and all these transactions are documented within the blockchain ledger. These records are public and transparent, which provides a secure and traceable mechanism for cold chain product security.
- Blockchain ledger. This ledger records the data-sharing transaction and related operations created during the cold chain products’ transportation. It is the only ledger of the BCCL system, and every cold chain institution retains a copy of this system’s blockchain ledger. When cold chain product disputes occurs, it can find the records about production, transportation, processing, testing, sales, etc. The transparency of records guarantees production security.

### 3.2. Data Sharing Transaction in BCCL System

**Figure 2:**
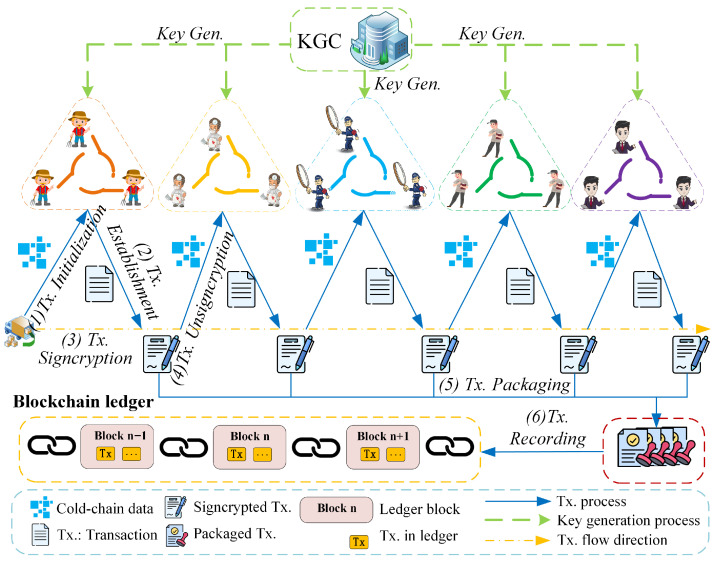
Data sharing transaction in BCCL system.

Through the TPP model, the cold chain logistics data is selected, shared, recorded, and protected. The data-sharing transaction (Tx.) includes six steps: Tx. initialization, Tx. establishment, Tx. signcryption, Tx. unsigncryption, Tx. packaging, and Tx. recording. A simple transaction example is shown in Figure 2.

(1) Tx. initialization. The operator in the production institution initiates a data-sharing transaction along with the cold chain product transportation process. He first obtains the transaction address from the BCCL system and prepares for this transaction. Here, the related production data and related operations are selected, which serve as the main transaction contents.
(2) Tx. establishment. The transaction operator uploads the production data into the data-sharing transaction and sends it to the next cold chain institution.
(3) Tx. signcryption. Before the sending process, this operator may sign this transaction with his private keys. Based on the LRSC scheme, the system performs both signing and encryption as a signcryption operation, which can effectively reduce the transaction time.
(4) Tx. unsigncryption. The receiver in the next cold chain institution first verifies its validity when he obtains the data-sharing transaction. He performs the unsigncryption operation where the legitimacy of the signature is verified and the transmitted message is decrypted at the same time.
(5) Tx. packaging. The cold chain institution acts as the system maintainer and competes with the bookkeeping rights of the blockchain ledger. Within a fixed period of time, these nodes all collect the published transactions in the BCCL system and package them into a block.
(6) Tx. recording. The nodes obtain accounting rights with a consensus protocol, such as proof of stake (PoS) or delegated proof of stake (DPoS). Then, the node that obtains accounting rights adds the newest block to the blockchain ledger.

Here, every cold chain institution performs step (1), step (2), step (3), and step (4) when the cold chain product passes through its operation. Meanwhile, all the transaction records about the same cold chain product will be packaged together. This mechanism can establish a complete record from production to consumption and forms the historic traceable process.

## 4. LRSC Scheme

### 4.1. Preliminaries

Several lattice-theoretic definitions relevant to the development of the LRSC scheme are presented in this section, which are regarded as the basis for its design and security analysis.

**Definition** **1** **(*****Lattice*** [43]**).** *Consider a finite collection of linearly independent vectors $\left\{b_{1},b_{2},\ldots ,b_{n}\right\}\subset R^{m}$. The collection of all integer linear combinations formed from these vectors,*

(1) $$\begin{array}{c} L=\left\{\sum_{i=1}^{n}z_{i}b_{i}\;|\;z_{i}\in Z\right\} \end{array}$$

*is called a lattice, denoted by L=L(B), where $B=[b_{1}\;|\;b_{2}\;|\;\ldots \;|\;b_{n}]\in R^{m\times n}$ is referred to as a basis of the lattice.*

**Definition** **2** **(*****q-ary Lattice*** [43]**).** *A q-ary lattice is associated with a prime modulus q and a matrix $\Lambda \in Z_{q}^{n\times m}$. Two related lattices are defined as follows:*

(2) $$\begin{array}{cc} \; & L^{⊥}\left(\Lambda \right)=\left\{x\in Z^{m}\;|\;\Lambda x=0\;\mathrm{mod}\;q\right\}\\ \; & L_{y}^{⊥}\left(\Lambda \right)=\left\{x\in Z^{m}\;|\;x=\Lambda^{T}y\;\mathrm{mod}\;q\;;\;y\in Z^{n}\right\} \end{array}$$

*Here, $L^{⊥}\left(\Lambda \right)$ is referred to as the dual lattice modulo q, and $L_{y}^{⊥}\left(\Lambda \right)$ represents a coset of $L^{⊥}\left(\Lambda \right)$ determined by a syndrome vector y.*

**Definition** **3** **(*****Gaussian distribution*** [44]**).** *Let L be a lattice within $Z^{m}$, let $c\in R^{m}$ be a center vector, and let s be a standard deviation. The discrete Gaussian distribution over L, centered at c with parameter s, denoted $D_{L,s,c}$, is the probability distribution that assigns to each point x∈L the probability:*

(3) $$\begin{array}{c} D_{L,s,c}\left(x\right)=\frac{\exp(-\frac{{∥x-c∥}^{2}}{2s^{2}})}{\sum_{y\in L}\exp\left(-\frac{{∥y-c∥}^{2}}{2s^{2}}\right)}=\frac{P_{s},c\left(x\right)}{P_{s},c\left(L\right)} \end{array}$$

*where $P_{s},c\left(x\right)$ is the Gaussian function centered at c, and the denominator ensures the distribution sums to one.*

**Definition** **4** **(*****Bimodal Gaussian distribution*** [44]**).** *Let L be a lattice within $Z^{m}$, and let s>0 be a standard deviation. Consider two center vectors $c_{1},c_{2}\in Z^{m}$ and a mixing coefficient ρ∈[0,1]. The generalized bimodal discrete Gaussian distribution over L is defined as a convex combination of two discrete Gaussian distributions centered at $c_{1}$ and $c_{2}$, respectively.*

Formally, the distribution $D_{L,s,c_{1},c_{2},\rho }$ is defined as:

(4) $$\begin{array}{c} D_{L,s,c_{1},c_{2},\rho }=\rho D_{L,s,c_{1}}\left(x\right)+\left(1-\rho \right)D_{L,s,c_{2}}\left(x\right)\;\mathrm{for}\;\mathrm{all}\;x\in L \end{array}$$

where each component distribution is given in Equation (3).

**Definition** **5** **(*Ring − SIS problem*** [45]**).** *Let $R=Z\left[x\right]/\langle f(x)\rangle$ be a cyclotomic ring for some monic polynomial f(x), let q∈Z be a modulus, and let $R_{q}=R/qR$ denote the polynomial ring modulo the polynomial f(x) and the integer q. Given k ring elements $a_{1},\ldots ,a_{k}\in R_{q}$, the Ring − SIS problem involves finding a non-zero tuple $z=\left(z_{1},\ldots ,z_{k}\right)\in R^{k}$ such that:*

(5) $$\begin{array}{c} \sum_{i=1}^{k}a_{i}z_{i}=0\;\;\mathrm{mod}\;q \end{array}$$

*where ${\left||z|\right|}_{2}\leq B$, and B>0 is the norm bound.*

**Definition** **6** **(*LWE and Decision − LWE problem*** [46]**).** *The LWE problem involves recovering a secret vector s given samples $\left(a_{i},b_{i}\right)$, where $a_{i}$ is uniformly random in $Z_{q}^{n}$, and $b_{i}$ equals the inner product of $a_{i}$ and s plus some noise $e_{i}$, modulo q. Meanwhile, the Decision-LWE problem requires deciding if the samples come from the LWE distribution or a uniform distribution over $Z_{q}^{n}\times Z_{q}$, where both components are independent and uniformly distributed.*

**Definition** **7** **(*****Gadget-Based Trapdoor Generation Algorithm*** [47]**).** *Given a security parameter n, a prime modulus q≥3, and an integer m≥nlogq, the gadget-based trapdoor generation algorithm GTrapGen(n,m,q)→(A,S) generates a matrix $A\in Z_{q}^{n\times m}$ composed as $A=[qI_{n}|A^{\prime }]$, where $I_{n}$ represents the identity matrix of dimension n, and $A^{\prime }\in Z_{q}^{n\times (m-n)}$ is sampled uniformly at random. The algorithm also outputs a trapdoor matrix $S\in Z^{m\times n}$ containing small elements such that:*

(6) $$\begin{array}{c} \mathrm{AS}=qI_{n}\;\mathrm{mod}\;q \end{array}$$

*The matrix S enables efficient sampling from the $L_{q}^{⊥}\left(\Lambda \right)=\left\{x\in Z^{m}\;|\;\Lambda x=0\;\mathrm{mod}\;q\right\}$ using discrete Gaussian techniques. Each column of S has an Euclidean norm limited by $\text{𝒪}(\sqrt{n\;\log\;q})$, and the trapdoor setup remains secure assuming the SIS problem over $Z_{q}$ is hard.*

### 4.2. Details of LRSC Scheme

This section presents a complete LRSC scheme, whose security relies on the lattice problem $R{\text{-SIS}}_{q,n,m,\beta }^{\kappa }$, which is defined in the ring R. To reduce computational overhead, the scheme employs **Z**${\text{-SIS}}_{q,n,m,\beta }^{\kappa }$, which provides equivalent post-quantum security. The scheme consists of four core algorithms: Setup, KeyGen, Signcryption algorithm, and Unsigncryption algorithm. The design and functionality of each algorithm are detailed as follows.

This section presents a full LRSC scheme composed primarily of four essential algorithms: Setup, Key Generation, Signcryption, and Unsigncryption. The specific descriptions of each are as follows. **Setup** $\left(1^{n}\right)$: The Key Generation Center (KGC) selects the security parameter $1^{n}$, a prime modulus *q*, a function $L=\text{𝒪}(\sqrt{n\log q})$ representing complexity, and positive integers m,d,k. It also defines a real-valued standard deviation $\sigma =12dk\sqrt{m}$ and a Gaussian width parameter $s=L\cdot \omega (\sqrt{\log n})$, guaranteeing m>2nlogq so that a trapdoor can be generated feasibly. The distribution $D_{\sigma }$ denotes a bimodal discrete Gaussian distribution with mean 0 and standard deviation σ. The pair $\left(E_{\partial }\left(\cdot \right),D_{\partial }\left(\cdot \right)\right)$ represents a symmetric encryption/decryption scheme, where the key *∂* is drawn from the key space ∑. The cryptographic hash functions below are specified according to Equation (7):

(7) $$\begin{array}{c} \left\{\begin{array}{c} H_{1}:{\left\{0,1\right\}}^{*}\to Z_{q}^{m}\\ H_{2}:{\left\{0,1\right\}}^{*}\to Z^{n}\\ H_{3}:{\left\{0,1\right\}}^{n}\to \sum \\ H_{4}:{\left\{0,1\right\}}^{*}\times {\left\{0,1\right\}}^{*}\to \Pi \end{array}\right. \end{array}$$

where Π denotes the ciphertext space.

Finally, the global public parameters $gp=\{H_{1},H_{2},H_{3},H_{4},q,n,m,d,k,\sigma ,s\}$ are output.

**KeyGen** (n,q,m): Algorithm 1 outlines the procedure for key generation. Given a security parameter $1^{n}$, a prime modulus q≥3, a positive integer m≥nlogq, and a ring of *r* participants, the KGC executes the gadget-based trapdoor generation algorithm GTrapGen (n,m,q). This procedure outputs a uniformly distributed random matrix $A_{i}\in Z_{q}^{n\times m}$ along with a compact trapdoor matrix $S_{i}\in Z_{q}^{m\times n}$ such that $A_{i}S_{i}=qI_{n}\;\left(\mathrm{mod}\;2q\right)$. The matrix $A_{i}$ is published as the public key of ring member *i*, while the matrix $S_{i}$ is securely kept as the corresponding private key of ring member *i*.
**Algorithm 1** KeyGen**Input:** 
$1^{n}$, *q*, *m*, *r***Output:** 
Public Key $A_{\left\{1,2,\ldots ,r\right\}}$ and Private Key $S_{\left\{1,2,\ldots ,r\right\}}$1:**for** i=1 to *r* **do**2:Generate $A_{i}\leftarrow \mathrm{GTrapGen}\left(n,m,q\right)$3:Compute $S_{i}\leftarrow A_{i}S_{i}=qI_{n}\;\left(\mathrm{mod}\;2q\right)$4:**end for**5:**return** $\left(A_{\left\{1,2,\ldots ,r\right\}},S_{\left\{1,2,\ldots ,r\right\}}\right)$

**Signcryption** $\left(A_{k},S_{k},\mu \right)$: In this phase, the signer’s identity in the ring $\{A_{1},\ldots ,A_{k},\ldots ,$$A_{r}\}$ is denoted by the public key $A_{k}$ of user *k*, serving as the basis for executing Algorithm 2.
**Algorithm 2** Signcryption**Input:** 
μ, $A_{k}$, $S_{k}$**Output:** 
ς  1:Compute $x=H_{1}\left(\sum_{i=1}^{r}A_{i}\right)$  2:Choose $y\leftarrow D_{\sigma }^{m}$  3:Compute λ=x+y  4:Compute $c\leftarrow H_{2}\left(A_{k}\lambda \;\left(\mathrm{mod}\;2q\right),\mu \right)$  5:Select b∈{0,1}  6:Compute $e\leftarrow \lambda +{\left(-1\right)}^{b}S_{k}c$  7:With probability $\min(\frac{D_{\sigma }^{m}\left(e\right)}{MD_{s,c\sigma }^{m}\left(e\right)},1)$, accept the vector *e* and output the signature (c,e)  8:Otherwise, **repeat Steps 1–8.**  9:Select $\tau \in {\left\{0,1\right\}}^{n}$ 10:Compute $z=E_{\partial =H_{3}\left(\tau \right)}\left(\mu ,c,e\right)$ 11:Compute $\eta =H_{4}\left(\tau ,z\right)$ 12:Choose ${\text{𝓁}}_{1},{\text{𝓁}}_{3}\leftarrow D_{\sigma }^{n},{\text{𝓁}}_{2}\leftarrow D_{\sigma }^{m}$ 13:Compute $v_{1}^{T}=-{\text{𝓁}}_{1}^{T}A_{k}+{\text{𝓁}}_{2}^{T}$ 14:Compute $v_{2}^{T}={\text{𝓁}}_{1}^{T}qI_{n}+{\text{𝓁}}_{3}^{T}+\tau \left\lfloor q/2\right\rfloor$ 15:**return** $\varsigma =(z,v_{1},v_{2})$

The signcryption process begins by binding the message μ to the signer through a signature. First, a hash value *x* is computed from the sum of the public keys of all ring members. Then, a vector *y* is generated according to a bimodal discrete Gaussian distribution to introduce randomness; this is combined with *x* to form an intermediate value λ. A challenge hash *c* is generated based on λ and the message μ. Using this challenge, a response vector *e* is computed with the involvement of the secret key. Finally, a rejection sampling step is applied before outputting the signature to ensure the correct statistical distribution and maintain security guarantees.

Next, to ensure both message confidentiality and signer anonymity, the signature is encrypted. A random bit string τ is selected, and the ciphertext *z* is generated by applying symmetric encryption $E_{\partial }$ to the message μ, signature hash *c*, and signature vector *e*,where the key $\partial =H_{3}\left(\tau \right)$ is derived from τ. Subsequently, a hash value η is computed to introduce pseudorandomness. Based on this hash, noise vectors ${\text{𝓁}}_{1},{\text{𝓁}}_{2}$ and ${\text{𝓁}}_{3}$, independently sampled from bimodal discrete Gaussian distributions, are used to construct auxiliary components $v_{1}$ and $v_{2}$. Together with the encrypted data *z*, these form the final ciphertext ς. This process ensures both security and resistance against attacks targeting anonymity and message confidentiality. Algorithm 2 illustrates the detailed steps.

**Unsigncryption** $\left(\varsigma ,A_{k},S_{k}\right)$: First, the ciphertext ς is decrypted to retrieve the original message μ along with its signature (c,e). An intermediate vector $\hat{\tau }$ is computed using components $v_{1}$ and $v_{2}$ and the private key $S_{k}$ from which the bitstring τ is derived. This bitstring τ is then used to acquire the symmetric decryption key *∂*, which decrypts the ciphertext component *z* to retrieve μ, *c*, and *e*.

Next, the signature (c,e) undergoes verification to ensure the message’s authenticity and integrity, as well as to ensure that the signer’s identity cannot be forged. This includes checking that the vector *e* satisfies certain norm constraints. If these conditions are met, the verification proceeds by validating the hash challenge *c* against the expected value computed using public information and the signature components. If the verification succeeds, the message is accepted; otherwise, the process outputs a rejection and terminates. The detailed procedure is shown in Algorithm 3.
**Algorithm 3** Unsigncryption**Input:** 
$\varsigma ,A_{k},S_{k}$**Output:** 
μ  1:Compute $\hat{\tau }=v_{1}^{T}S_{k}+v_{2}^{T}$  2:Let $\hat{\tau }=\left(\tau_{1}^{\prime },\tau_{2}^{\prime },\ldots ,\tau_{\delta }^{\prime }\right)$  3:**for** i=1 to δ **do**  4:**if** $\tau_{i}^{\prime }\in \left(-\left\lfloor q/4\right\rfloor ,\left\lfloor q/4\right\rfloor \right)$ **then**  5:   $\tau_{i}=0$  6:**else**  7:   $\tau_{i}=1$  8:**end if**  9:**return** $\tau =(\tau_{1},\tau_{2},\ldots ,\tau_{\delta })$ 10:Compute $D_{\partial =H_{3}\left(\tau \right)}\left(z\right)=\left(\mu ,c,e\right)$ 11:**if** $∥e∥\leq B_{2},{∥e∥}_{\infty }\leq q/4$ **then** 12:   **terminate** 13:**else** 14:   **continue to the next step** 15:**end if** 16:Compute $c^{\prime }=H_{2}\left(A_{k}e+qc\;\left(\mathrm{mod}\;2q\right),\mu \right)$ 17:**if** $c^{\prime }=c$ **then**
 18:   **return** μ 19:**else** 20:   **return** ⊥ 21:**end if**

The proposed LRSC scheme integrates the signature and encryption operations into a single process, thereby ensuring both the confidentiality of the message and the anonymity of the signer while significantly reducing the computational and communication overhead of the system.

## 5. Security Analysis

This section offers a comprehensive examination of the correctness of the proposed scheme and presents formal security proofs under the IND-CCA (Indistinguishability under Chosen Ciphertext Attack) and UF-CMA (Unforgeability under Chosen Message Attack) security models.

### 5.1. Correctness

To verify the correctness of bit recovery from the noisy encoding $\hat{\tau }$, observe that, according to Equation (8), $\hat{\tau }$ is computed as: $\hat{\tau }={\text{𝓁}}_{2}^{T}S_{k}+{\text{𝓁}}_{3}^{T}+\tau \left\lfloor q/2\right\rfloor$, where ${\text{𝓁}}_{2},{\text{𝓁}}_{3}$ represents a minor noise component drawn from a discrete Gaussian distribution. If $\hat{\tau }\in \left(-\left\lfloor q/4\right\rfloor ,\left\lfloor q/4\right\rfloor \right)$, the bit is decoded as τ=0; otherwise, it is closer to q/2, and the bit is decoded as τ=1.

(8) $$\begin{array}{cc} \; & \hat{\tau }=v_{1}^{T}S_{k}+v_{2}^{T}\\ \; & \;\;=\left(-{\text{𝓁}}_{1}^{T}A_{k}+{\text{𝓁}}_{2}^{T}\right)S_{k}+{\text{𝓁}}_{1}^{T}qI_{n}+{\text{𝓁}}_{3}^{T}+\tau \left\lfloor q/2\right\rfloor \\ \; & \;\;=-{\text{𝓁}}_{1}^{T}A_{k}S_{k}+{\text{𝓁}}_{2}^{T}S_{k}+{\text{𝓁}}_{1}^{T}qI_{n}+{\text{𝓁}}_{3}^{T}+\tau \left\lfloor q/2\right\rfloor \\ \; & \;\;={\text{𝓁}}_{2}^{T}S_{k}+{\text{𝓁}}_{3}^{T}+\tau \left\lfloor q/2\right\rfloor \end{array}$$

To verify the correctness of the tag $c^{\prime }$, i.e., to confirm that $c^{\prime }=c$, one can check whether the following equation holds: $A_{k}e+qc\;\left(\mathrm{mod}\;2q\right)=A_{k}\lambda \;\left(\mathrm{mod}\;2q\right)$. Since $e=\lambda +{\left(-1\right)}^{b}S_{k}c$, after the derivation of Equation (9), it is proven that this equation holds; that is, $c^{\prime }=c$ is satisfied.

(9) $$\begin{array}{c} \left\{\begin{array}{c} A_{k}e+qc\;\left(\mathrm{mod}\;2q\right)=A_{k}\left(\lambda +{\left(-1\right)}^{b}S_{k}c\right)+qc\;\left(\mathrm{mod}\;2q\right)=A_{k}\lambda \;\left(\mathrm{mod}\;2q\right)\\ H_{2}\left(A_{k}e+qc\;\left(\mathrm{mod}\;2q\right),\mu \right)=c^{\prime }=c=H_{2}\left(A_{k}\lambda \;\left(\mathrm{mod}\;2q\right),\mu \right) \end{array}\right. \end{array}$$

### 5.2. Confidentiality

**Theorem** **1.** *Assume there is a probabilistic polynomial-time attacker A who can compromise the IND-CCA2 security of the proposed LRSC scheme with a significant probability ϵ. Then, there is a probabilistic polynomial-time algorithm C capable of solving the* ***Decision − LWE problem*** *with a significant probability $\epsilon^{\prime }$.*

**Proof.**  Algorithm C is constructed to address the **Decision − LWE problem**. Its goal is, given a Decisional − LWE instance, to decide if the given sample originates from the LWE distribution or is uniformly random. This determination is accomplished by employing adversary A within algorithm C as a subprocedure. During this process, C maintains seven lists, namely list1 to list7, which are used to record the results of random oracle queries, including hash function queries, public–private key queries, and signcryption queries. Then, C selects an identity $id_{t}$ from the adversary’s queries to the hash function $H_{1}$ to serve as the challenge identity, and it embeds the LWE instance in its public key. When A successfully distinguishes the signcryption challenge with a certain probability, C is able to solve the **Decision − LWE problem** with an equivalent probability.

During the security game’s initialization, challenger C runs the Setup algorithm to generate the global public parameters gp, which are then sent to the adversary A. Under an adaptive model, adversary A can issue a polynomially bounded number of queries to challenger C, including hash queries, public key and private key requests, and signcryption queries.

**$H_{1}$ Query**: Upon receiving a hash query for user identity $id_{i}$ from adversary A, challenger C performs the following steps.

- If the pair $\left(id_{i},x\right)$ is found in list1, C returns the stored hash value $x=H_{1}\left(\sum_{i=1}^{r}A_{i}\right)$ to A;
- Otherwise, C samples a vector $x\leftarrow Z_{q}^{m}$ uniformly at random, stores $\left(id_{i},x\right)$ in list1, and returns *x* to A.

**$H_{2}$ Query**: When the adversary A requests the hash value corresponding to the tuple $\left(A_{i},\lambda ,\mu \right)$, the challenger C verifies whether the list list2 contains an entry $\left(A_{i},\lambda ,\mu ,c,e\right)$.

- If such an entry is found, C returns the values (c,e) to A;
- Otherwise, C samples $e\leftarrow D_{\sigma }^{m}$ from the Gaussian distribution and chooses $c\leftarrow Z^{n}$ uniformly at random. The challenger C then returns (c,e) to the adversary A and records the new entry $\left(A_{i},\lambda ,\mu ,c,e\right)$ in list2.

**$H_{3}$ Query**: Upon receiving a query for $H_{3}\left(\tau \right)$, the challenger C verifies whether the pair $\left(\tau ,H_{3}\left(\tau \right)\right)$ is already stored in the list list3.

- If the pair exists, the corresponding symmetric key $\partial =H_{3}\left(\tau \right)$ is returned to A;
- Otherwise, C samples ∂←∑ uniformly at random from the key space ∑, sets $H_{3}\left(\tau \right)=\partial$, returns *∂* to A, and records the mapping $\left(\tau ,H_{3}\left(\tau \right)\right)$ into list3.

**$H_{4}$ Query**: Upon receiving a query for $H_{4}\left(\tau ,z\right)$, the challenger C verifies whether the tuple (τ,z,η) is present in the list list4.

- If such an entry exists, the corresponding hash value η is returned to A;
- Otherwise, C randomly samples η←Π from the coin-flipping-based output space Π, sets $H_{4}\left(\tau ,z\right)=\eta$, returns η to A, and records (τ,z,η) into list4.

**Public Key Query**: Upon receiving a query for user *i*’s public key $A_{i}$ from adversary A, challenger C first checks whether $A_{i}$ is already stored in list5.

- If so, it returns the corresponding $A_{i}$ to A;
- Otherwise, C uniformly samples a matrix $A_{i}\in Z_{q}^{n\times m}$, records it in list5, and returns $A_{i}$ to A.

**Private Key Query**: When adversary A requests user *i*’s private key $S_{i}$, the challenger C first checks whether the queried identity $id_{i}$ matches the challenge identity $id_{t}$.

- If $id_{i}=id_{t}$, C aborts the game;
- Otherwise, it computes the private trapdoor matrix $S_{i}$ satisfying $A_{i}S_{i}=qI_{n}\;\left(\mathrm{mod}\;2q\right)$, stores the tuple $\left(id_{i},A_{i},S_{i}\right)$ in list6, and returns $S_{i}$ to A.

**Signcryption Query**: When the adversary A requests the signcryption ciphertext corresponding to identity $id_{i}$ and message μ, the challenger C first checks whether $id_{i}$ equals the challenge identity $id_{t}$.

- If $id_{i}\neq id_{t}$, C executes the Algorithm 2 signcryption and returns the resulting ciphertext ς to A;
- Otherwise, C retrieves the key pair $\left(A_{i},S_{i}\right)$ from list7 and proceeds to simulate the signcryption process as follows:
  - Computes $x=H_{1}\left(\sum_{i=1}^{r}A_{i}\right)$;
  - Computes λ=x+y;
  - Computes $c\leftarrow H_{2}\left(A_{k}\lambda \;\left(\mathrm{mod}\;2q\right),\mu \right)$;
  - Computes $e\leftarrow \lambda +{\left(-1\right)}^{b}S_{k}c$;
  - Computes $z=E_{\partial =H_{3}\left(\tau \right)}\left(\mu ,c,e\right)$;
  - Computes $\eta =H_{4}\left(\tau ,z\right)$;
  - Computes $v_{1}^{T}=-{\text{𝓁}}_{1}^{T}A_{k}+{\text{𝓁}}_{2}^{T}$;
  - Computes $v_{2}^{T}={\text{𝓁}}_{1}^{T}qI_{n}+{\text{𝓁}}_{3}^{T}+\tau \left\lfloor q/2\right\rfloor$;
  - Returns ciphertext ς to A.

**Unsigncryption Query**: When adversary A requests the unsigncryption of ciphertext ς linked to identity $id_{i}$, the challenger C proceeds as follows:

- If $id_{i}\neq id_{t}$, C performs the standard unsigncryption algorithm and returns the result to A.
- Otherwise, C simulates the process by:
  - Computing $\hat{\tau }=v_{1}^{T}S_{k}+v_{2}^{T}$, and for each component $\tau_{i}^{\prime }\in \hat{\tau }$, setting
    (10) $$\tau_{i}=\left\{\begin{array}{c} 0\;\;\mathrm{if}\;\tau_{i}^{\prime }\in \left(-\left\lfloor q/4\right\rfloor ,\left\lfloor q/4\right\rfloor \right),\\ 1\;\;\mathrm{otherwise}. \end{array}\right.$$
  - Computing $D_{\partial }\left(z\right)=\left(\mu ,c,e\right)$;
  - Verifying whether $∥e∥\leq B_{2}$ and ${∥e∥}_{\infty }\leq q/4$;
  - Querying the random oracle $H_{2}$ to get $c^{\prime }=H_{2}\left(A_{k}e+qc\;\left(\mathrm{mod}\;2q\right),\mu \right)$; if $c=c^{\prime }$, then μ is accepted as a valid message; otherwise, ⊥ is output.

Then, challenger C receives the message $\mu_{b}$ and identity $id_{i}^{*}$ from the adversary A, where b∈{0,1}. Prior to the start of the game, querying the private key of $id_{i}^{*}$ or replacing its public key by the adversary is not allowed. If $id_{i}^{*}\neq id_{t}$, C aborts the game; otherwise, it proceeds with the following simulation steps:

- Computes $x=H_{1}\left(\sum_{i=1}^{r}A_{i}\right)$;
- Selects $y^{*}\leftarrow D_{\sigma }^{m}$, computes $\lambda^{*}=x+y^{*}$;
- Computes $c^{*}\leftarrow H_{2}\left(A_{k}\lambda^{*}\;\left(\mathrm{mod}\;2q\right),\mu_{b}\right)$;
- Computes $e^{*}\leftarrow \lambda^{*}+{\left(-1\right)}^{b}S_{k}c^{*}$;
- Computes $z^{*}=E_{\partial =H_{3}\left(\tau \right)}\left(\mu ,c^{*},e^{*}\right)$;
- Computes $\eta =H_{4}\left(\tau ,z^{*}\right)$;
- Computes $v_{1}^{T}=-{\text{𝓁}}_{1}^{T}A_{k}+{\text{𝓁}}_{2}^{T}$;
- Computes $v_{2}^{T}={\text{𝓁}}_{1}^{T}qI_{n}+{\text{𝓁}}_{3}^{T}+\tau \left\lfloor q/2\right\rfloor$;
- Returns ciphertext $\varsigma^{*}=\left(z^{*},v_{1}^{*},v_{2}^{*}\right)$ to A.

After the challenge phase, the adversary A is authorized to make a limited number of additional queries, bounded by a polynomial function. Throughout the entire game, A is prohibited from obtaining the private key corresponding to the challenge identity $id_{i}^{*}$ or replacing its public key prior to the challenge phase. Moreover, once the challenge ciphertext $\varsigma^{*}$ is received, the adversary is prohibited from querying the unsigncryption oracle on $\varsigma^{*}$. These constraints ensure that the adversary cannot gain an unfair advantage and preserve the integrity of the IND-CCA2 security game.

Finally, the challenger C receives the adversary A guess $b^{\prime }$ for the challenge bit *b*. If $b=b^{\prime }$, this indicates that the adversary has successfully distinguished the real message bound to the challenge ciphertext. Consequently, C learns that the private key matrix $S_{i}^{*}\in Z^{m\times n}$ and the public key matrix $A_{i}^{*}\in Z^{n\times m}$ have all their entries bounded by 7σ, and the following key generation equation is satisfied: $A_{i}^{*}S_{i}^{*}=qI_{n}\;\left(\mathrm{mod}\;2q\right)$. Otherwise, if $b\neq b^{\prime }$, then $A_{i}^{*}$ is concluded to be drawn uniformly at random. Therefore, algorithm C successfully distinguishes between the two distributions in the decisional − LWE problem, completing the reduction and establishing that the IND-CCA security of the proposed scheme relies on the computational difficulty of the decisional − LWE assumption.

In the game, the probability of correctly guessing the challenge bit *b* is at most 1/2. Regardless of how many queries the adversary makes, the success probability for distinguishing the value of *b* remains negligible. Furthermore, the probability of solving the decisional − LWE problem is given by $\epsilon^{\prime }=\frac{\epsilon }{q_{1}+2q_{2}+2q_{3}+\frac{3}{2}q_{4}+q_{pk}+q_{sk}+q_{sc}+q_{unsc}}$, where $q_{i=\{1,2,3,4\}}$ represent the number of queries made to the hash functions $H_{i}$, with $q_{pk}$, $q_{sk}$, $q_{sc}$, and $q_{unsc}$ denoting the counts of public key, private key, signcryption, and unsigncryption queries, respectively. If no efficient algorithm exists that can solve the decisional LWE problem with non-negligible advantage, then the assumption that an adversary A can break the IND-CCA security of the LRSC scheme with advantage ϵ is invalid. Thus, under the hardness of the decisional LWE problem, the proposed LRSC scheme is IND-CCA secure. □

### 5.3. Unforgeability

**Theorem** **2.** *Suppose there is an adversary A capable of successfully attacking the UF-CMA security of the proposed LRSC scheme with a significant advantage ϵ. Then, it is possible to construct a reduction algorithm C, which, by leveraging A as a subroutine, one can resolve the SIS problem under condition $\epsilon^{\prime }$ with non-negligible success probability.*

**Proof.**  The challenger C is given a randomly selected instance of the SIS problem, where the task is to identify a non-zero short vector $\upsilon \in Z^{m}$ that satisfies Aυ=0(modq) for a given matrix $A\in Z_{q}^{n\times m}$. Equivalently, υ serves as a solution to the given SIS instance.

In the initial phase of the game, the adversary A receives the global public parameters gp generated by challenger C by running the **Setup** $\left(1^{n}\right)$ procedure. Subsequently, A is permitted to adaptively issue the same types of queries described in **Theorem 1**, including queries to hash functions $H_{i}$ for i=1,2,3,4, as well as public/private key queries, signcryption queries, and unsigncryption queries.

In the game, if A is capable of producing a valid signature $\left(c^{*},e^{*}\right)$ for an arbitrary message $\mu^{*}$ based on the ciphertext, then A outputs a forged ciphertext $\varsigma^{*}$. If $id_{i}^{*}\neq id_{t}$, the challenger C aborts the game. Otherwise, the adversary forges another ciphertext $\varsigma^{**}$ and its corresponding signature $\left(c^{**},e^{**}\right)$. Since the hash value is computed as $c\leftarrow H_{2}\left(A_{k}\lambda \;\left(\mathrm{mod}\;2q\right),\mu \right)$ and the message μ is the same, it follows that $c^{*}=c^{**}$.

From Equation (11):

(11) $$\begin{array}{c} \left\{\begin{array}{c} A_{k}e^{*}+qc^{*}=A_{k}\lambda^{*}\\ A_{k}e^{**}+qc^{**}=A_{k}\lambda^{**} \end{array}\right. \end{array}$$

Subtracting the two gives:

(12) $$\begin{array}{c} A_{k}\left(e^{*}-e^{**}+\lambda^{**}-\lambda^{*}\right)=q\left(c^{**}-c^{*}\right)\;\left(\mathrm{mod}\;2q\right) \end{array}$$

Given $c^{**}=c^{*}$, the right-hand side is zero:

(13) $$\begin{array}{c} A_{k}\left(e^{*}-e^{**}+\lambda^{**}-\lambda^{*}\right)=0 \end{array}$$

Let $\upsilon =e^{*}-e^{**}+\lambda^{**}-\lambda^{*}$. Then, $A_{k}\upsilon =0\;\left(\mathrm{mod}\;q\right)$, which implies that υ is a solution to the SIS problem.

As long as $∥\upsilon ∥\neq 0$, i.e., $e^{*}\neq e^{**}$ or $\lambda^{**}\neq \lambda^{*}$, the adversary has found a non-zero short vector υ, thereby solving the SIS problem. This contradicts the SIS assumption, meaning that the adversary A cannot forge valid signatures or derive the private key.

In the game, the challenger’s advantage in solving the SIS problem is expressed as $\epsilon^{\prime }=\frac{\epsilon }{q_{1}+2q_{2}+2q_{3}+\frac{3}{2}q_{4}+q_{pk}+q_{sk}+q_{sc}+q_{unsc}}$, where each signcryption and unsigncryption query contributes one $H_{2}$ query, one $H_{3}$ query, and half of an $H_{4}$ query. Since the total number of queries grows at most polynomially and the advantage is evenly distributed across them, $\epsilon^{\prime }$ is negligible. In practical terms, if the SIS problem is hard to solve under current computational assumptions, then no probabilistic polynomial-time adversary can produce a valid signature. Therefore, the proposed scheme achieves security under the UF-CMA model. □

## 6. Comparison and Performance

To further evaluate the computational efficiency of the LRSC scheme, this section presents two sets of simulation experiments. The first focuses on comparing the sizes of keys and ciphertexts, while the second measures the time consumption of the signcryption and unsigncryption operations. All tests were carried out using a device with a laptop and 16GB of RAM, using MATLAB 2021b as the simulation platform.

### 6.1. Comparison of Key and Ciphertext Sizes

In this section, the comparison centers on evaluating the LRSC scheme against existing similar schemes based on the sizes of keys, signatures, and ciphertexts. According to the structure of these schemes, their performance typically depends heavily on the lengths of public/private keys and ciphertexts. Based on analytical research, the public/private key sizes, signature sizes, and ciphertext sizes of the LRSC scheme and those in references [37,38,39,40,41] are summarized. The relevant data are shown in Table 1. As shown in the table, the LRSC scheme features smaller signature and ciphertext sizes, which reduce communication and storage overhead, making it suitable for resource-constrained environments. Although the key sizes are larger, they are used mainly during initialization and thus have a limited impact while offering stronger resistance against quantum attacks and enhancing overall security.

To present size differences among various schemes more clearly, simulations were conducted using the parameters specified in Table 2. Among these parameters, the value of parameter *n* corresponds to three security levels (128-bit, 160-bit, and 192-bit) and is selected in conjunction with modulus *q* to ensure the post-quantum security of the system; the values of parameters *m* and *k* strictly adhere to lattice-based dimension constraints, ensuring that the SIS problem meets the hardness requirement within a given range; parameter *r* represents the ring size, which is set to 3, 5, and 10, respectively, to compare the performance differences of the scheme under different ring sizes.

**Figure 3:**
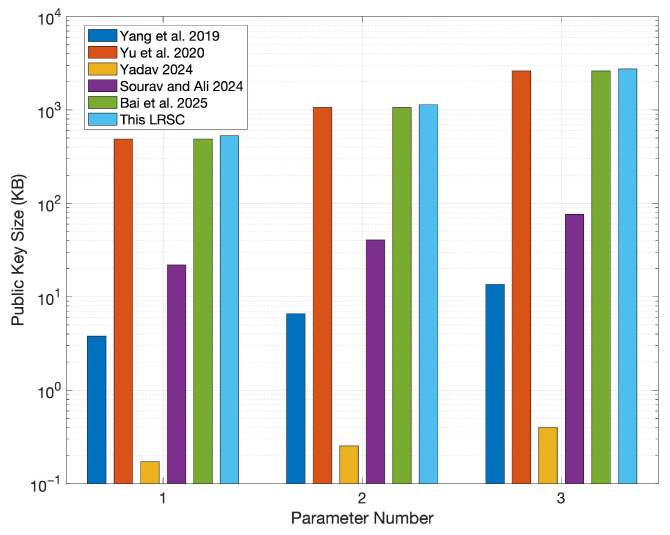
Comparison of public key sizes [37,38,39,40,41].

**Figure 4:**
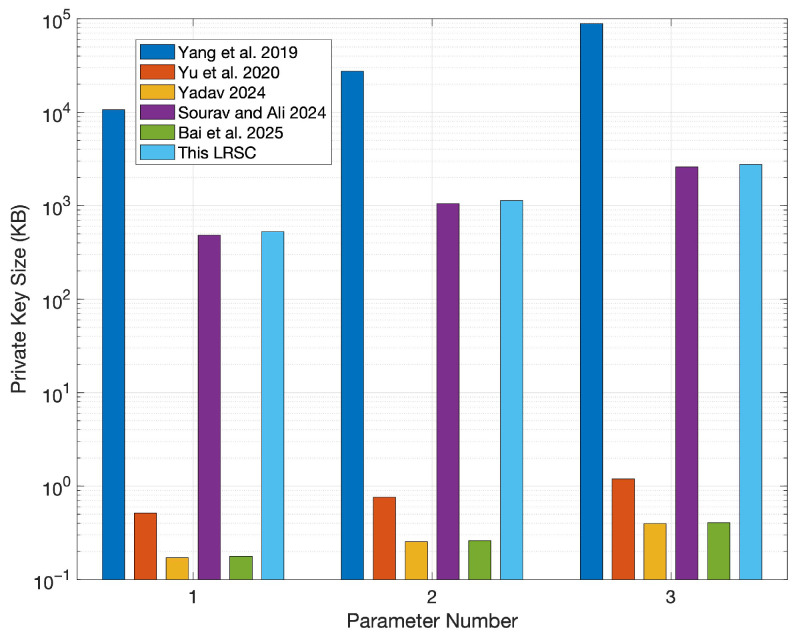
Comparison of private key sizes [37,38,39,40,41].

**Figure 5:**
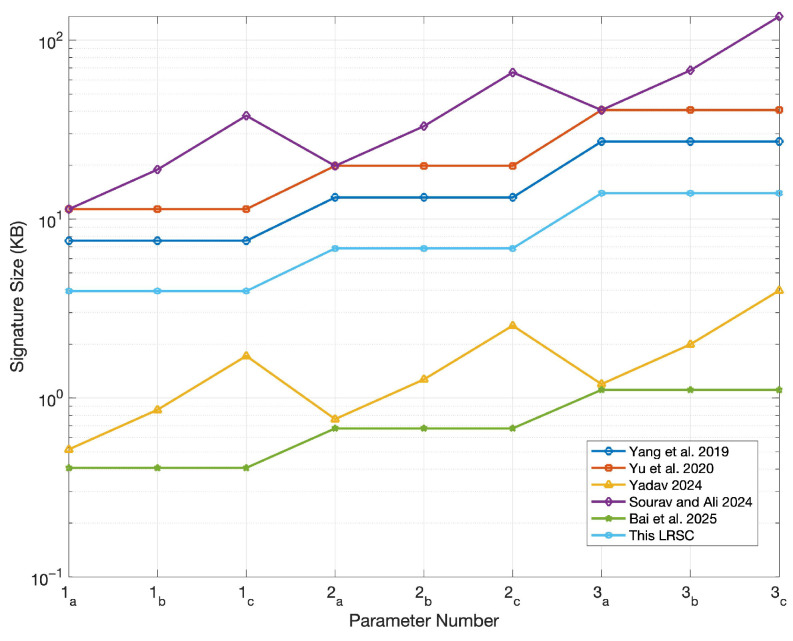
Comparison of signature sizes [37,38,39,40,41].

**Figure 6:**
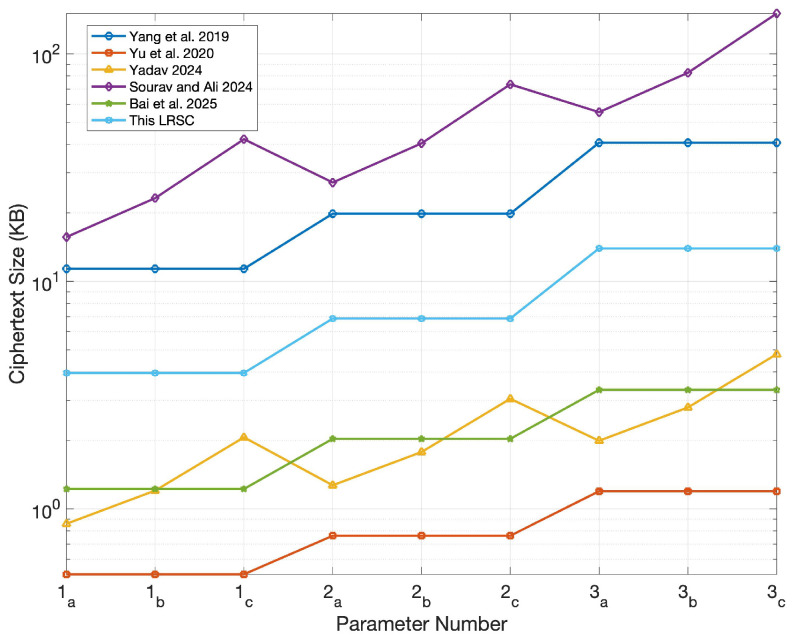
Comparison of ciphertext sizes [37,38,39,40,41].

Furthermore, to clearly distinguish the independent impacts of “different parameters” and “different ring sizes” on system performance and security, the experiment was further designed with three categories of parameter groups (labeled as 1, 2, and 3 in sequence). Each parameter group is subdivided into three subcategories (a, b, and c)—this subdivision is intended to independently examine the specific impact of ring size variations on system performance under the same parameter configuration. The relevant experimental results are shown in Figure 3, Figure 4, Figure 5 and Figure 6.

Figure 3 demonstrates that, under different parameter settings, the public key size of the proposed scheme remains at a comparable level to those of the schemes in references [38,41], indicating that its storage overhead for key generation is consistent with mainstream approaches. The scheme in reference [39], however, achieves the smallest public key size. While this offers an advantage in terms of storage efficiency, such a design typically entails increased computational complexity or reduced flexibility, making it less universally applicable.

As shown in Figure 4, the private key size of the proposed scheme is close to that of the scheme in reference [40], suggesting that both employ similar structural optimization strategies in private key construction. The schemes in references [39,41] present the smallest private keys, which enhances portability. Nevertheless, this advantage must be weighed against their security strength and key generation efficiency, as excessive optimization could compromise overall security.

Figure 5 shows that the proposed scheme achieves a relatively small signature size, second only to the schemes in references [39,41]. Furthermore, its signature size remains stable regardless of variations in ring size. This insensitivity to ring size enhances the scheme’s scalability—given that a larger ring size typically corresponds to higher security requirements. In contrast, the signature sizes of the scheme in reference [39] (which has a smaller signature size than the proposed scheme) and the scheme in reference [40] increase as the ring size grows, indicating potential limitations for their large-scale deployment. While the scheme in reference [41] reduces the private key size, it sacrifices signature compactness, which may lead to increased transmission costs. Overall, the proposed scheme achieves a balanced trade-off among security, efficiency, and scalability.

Finally, Figure 6 shows that the ciphertext size of the proposed scheme remains at an intermediate level and does not change as the ring size increases. This stability enhances the scheme’s scalability under high-security parameters. In contrast, the ciphertext sizes of the schemes in references [39,40] increase significantly with the growth of ring size, which limits their efficiency in large-scale applications. Although the ciphertext size of the proposed scheme is not the smallest, this design avoids the additional computational overhead caused by excessive compression and achieves a reasonable balance among security, efficiency, and resource consumption.

Overall, these comparative results indicate that the proposed scheme maintains a comparable level with mainstream schemes in terms of public and private key sizes while demonstrating a clear advantage in signature size, thereby highlighting its superior communication efficiency and deployment value in practical applications.

### 6.2. Performance Evaluation

In the performance evaluation, the primary focus is on the time overhead of signcryption and unsigncryption, as presented in Table 3. A lower time overhead reflects higher computational efficiency, which is especially beneficial for environments with limited resources, like mobile devices and IoT systems. Additionally, faster signcryption and unsigncryption processes contribute to improved system responsiveness, enhanced user experience, and better scalability in practical deployments. According to Table 3, the performance of the schemes in [39,40] is significantly affected by the number of ring members, whereas the schemes in [37,38,41], as well as the proposed scheme, maintain consistent performance irrespective of ring size. Notably, both [38] and the proposed scheme exhibit relatively low time consumption, underscoring their efficiency and practicality in real-world applications.

**Figure 7:**
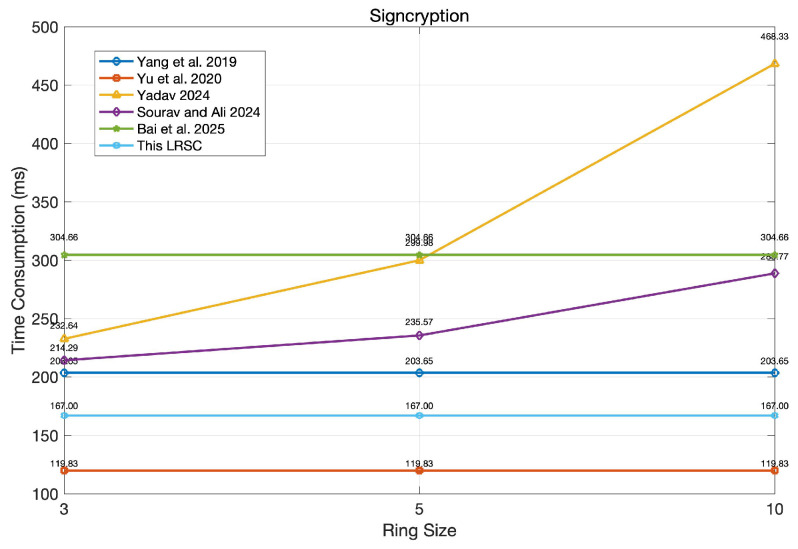
Comparison of signcryption running time [37,38,39,40,41].

**Figure 8:**
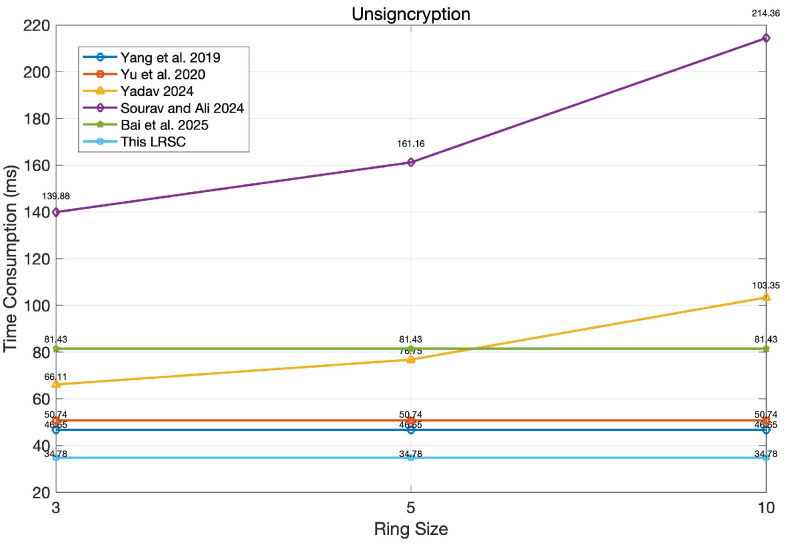
Comparison of unsigncryption running time [37,38,39,40,41].

To clearly observe the differences among various schemes, this study conducted simulation experiments based on the algorithm runtimes listed in Table 4. Each runtime represents the average value obtained after 200 iterations performed under the same conditions, and the results are presented in Figure 7 and Figure 8 (with the ring size set to r = 3, 5, 10).

As shown in the Figure 7, the signcryption time of the proposed scheme remains stable at 167 ms and does not change with the ring size, second only to the 119.83 ms of reference [38]. This indicates that the proposed scheme reduces its dependence on the ring size through design optimization during the signcryption phase, thereby ensuring good scalability. Although the scheme in reference [38] operates faster, its advantage may stem from structural simplification, which might involve certain trade-offs in terms of security or flexibility.

As shown in Figure 8, during the unsigncryption phase, the average time consumption of the proposed scheme is 34.78 ms, the shortest among all compared schemes. This result not only demonstrates the high efficiency of the unsigncryption algorithm design but also indicates that the scheme has significant advantages in scenarios with high real-time requirements. In contrast, other schemes exhibit varying degrees of delay in unsigncryption time, which may affect the response speed in large-scale concurrent environments.

Overall, while ensuring stable signcryption speed, the proposed scheme achieves significant optimization in the unsigncryption phase, reflecting its balanced design among security, scalability, and computational efficiency. The research results show that the scheme has stronger practicality in the BCCL context, and it is particularly suitable for complex application environments that demand extremely high efficiency and fast response.

## 7. Conclusions

Facing the security issues of BCCL systems, this paper introduces a TPP model and an LRSC scheme for cross-institutional logistics data sharing. The TPP model can protect the real signer’s privacy, and the signcryption mechanism can support the simultaneous operations of signature and encryption. This LRSC scheme can support the TPP model to guarantee privacy and security in logistics data-sharing processes. At the same time, the lattice assumption strengthens the quantum-resistant security of BCCL systems. Moreover, the security proof and experiment findings confirm the security and efficiency of the proposed TPP model and LRSC scheme. In addition, with the expansion of logistics devices, device authentication, cross-device data sharing, and efficient transaction search are the hottest research areas in future work.

## Figures and Tables

**Table 1:** Key size comparison.

Schemes	Public Key	Private Key	Signature	Ciphertext
Yang et al. [37]	mlogq	mmlogq	2mlogq	(2+3m)logq
Yu et al. [38]	m(1+n)logq	3nlogq	(3m+logq)logq	3nlogq
Yadav [39]	nlogq	nlogq	rnlogq	(2+r)nlogq
Sourav and Ali [40]	$n^{2}\log q$	mnlogq	rmlogq	(3n+(r+1)m)logq
Bai et al. [41]	((n+1)m+1)logq	(4+n)logq	(2+n+k)logq	(8+3n+3k)logq
This LRSC	mnlog2q	mnlog2q	(m+n)logq	(n+m)logq

**Table 2:** Parameter settings.

Number	1			2			3		
	** $1_{a}$ **	** $1_{b}$ **	** $1_{c}$ **	** $2_{a}$ **	** $2_{b}$ **	** $2_{c}$ **	** $3_{a}$ **	** $3_{b}$ **	** $3_{c}$ **
*n*	128	128	128	160	160	160	192	192	192
*q*	$2^{11}$	$2^{11}$	$2^{11}$	$2^{13}$	$2^{13}$	$2^{13}$	$2^{17}$	$2^{17}$	$2^{17}$
*m*	2818	2818	2818	4164	4164	4164	6533	6533	6533
*k*	173	173	173	264	264	264	341	341	341
*r*	3	5	10	3	5	10	3	5	10

**Table 3:** Comparison of running time.

Schemes	Signcryption	Unsigncryption
Yang et al. [37]	$6T_{M}+3T_{H}+4T_{D}+T_{I}$	$6T_{M}+T_{H}$
Yu et al. [38]	$4T_{M}+2T_{H}+3T_{D}$	$4T_{M}+2T_{H}$
Yadav [39]	$2rT_{M}+T_{H}+\left(r+2\right)T_{D}+2T_{I}$	$rT_{M}+T_{H}+T_{I}$
Sourav and Ali [40]	$\left(2r+2\right)T_{M}+4T_{D}+3T_{H}+T_{I}$	$\left(2r+1\right)T_{M}+T_{D}+3T_{H}+T_{I}$
Bai et al. [41]	$10T_{M}+3T_{H}+9T_{D}$	$7T_{M}+3T_{H}$
This LRSC	$3T_{M}+4T_{D}+4T_{H}$	$T_{M}+2T_{H}$

**Table 4:** The average running time of each algorithm.

Operation	Time Consumption (Times/ms)	Algorithm Description
$T_{M}$	5.32 ms	Matrix–vector multiplication
$T_{H}$	14.73 ms	Hash function
$T_{D}$	23.03 ms	Gaussian sampling
$T_{I}$	35.42 ms	Image sampling

## Data Availability

No new data were created or analyzed in this study. Data sharing is not applicable to this article.

## References

[1] Yu M., Zhang H., Ma J., Duan X., Kang S., Li J. (2024). Cold chain logistics supervision of agricultural products supported using internet of things technology. IEEE Internet Things J..

[2] Al-Farsi S., Rathore M.M., Bakiras S. (2021). Security of blockchain-based supply chain management systems: Challenges and opportunities. Appl. Sci..

[3] Hu X. (2022). Cold chain logistics model of agricultural products based on embedded system and blockchain. Prod. Plan. Control.

[4] Song H., Vajdi A., Wang Y., Zhou J. (2021). Blockchain for consortium: A practical paradigm in agricultural supply chain system. Expert Syst. Appl..

[5] He M., Wang H., Sun Y., Bie R., Lan T., Song Q., Zeng X., Pustisĕk M., Qiu Z. (2022). T2L: A traceable and trustable consortium blockchain for logistics. Digit. Commun. Netw..

[6] Allenbrand C. (2023). Smart contract-enabled consortium blockchains for the control of supply chain information distortion. Blockchain Res. Appl..

[7] Zhang Y., Tang Y., Li C., Dong M., Huang M., Zhang H., Ota K. (2024). Privacy-preserving for blockchain-enabled cold-chain logistics system with IoV and linkable ring signature. IEEE Trans. Veh. Technol..

[8] Xiong R., Cheng J., Dong X., Pu J., Shan F. (2025). Leveraging consortium blockchain for secure cross-domain data sharing in supply chain networks. IEEE Trans. Serv. Comput..

[9] Kim S., Kim J., Kim D. (2020). Implementation of a blood cold chain system using blockchain technology. Appl. Sci..

[10] Zhang X., Sun Y., Sun Y. (2022). Research on cold chain logistics traceability system of fresh agricultural products based on blockchain. Comput. Intell. Neurosci..

[11] Yang R., Wakefield R., Lyu S., Jayasuriya S., Han F., Yi X., Yang X., Amarasinghe G., Chen S. (2020). Public and private blockchain in construction business process and information integration. Autom. Constr..

[12] Si Y. (2022). Agricultural cold chain logistics mode based on multi-mode blockchain data model. Comput. Intell. Neurosci..

[13] Bottoni P., Di Ciccio C., Pareschi R., Tortola D., Gessa N., Massa G. (2023). Blockchain-as-a-service and blockchain-as-a-partner: Implementation options for supply chain optimization. Blockchain: Res. Appl..

[14] Gao S., Zhang Z., Li Q., Ding S., Iu H.H.C., Cao Y., Xu X., Wang C., Mou J. (2025). Encrypt a story: A video segment encryption method based on the discrete sinusoidal memristive rulkov neuron. IEEE Trans. Dependable Secur. Comput..

[15] Gao S., Wu R., Iu H.H.C., Erkan U., Cao Y., Li Q., Toktas A., Mou J. (2025). Chaos-based video encryption techniques: A review. Comput. Sci. Rev..

[16] Cha S., Baek S., Kim S. (2020). Blockchain based sensitive data management by using key escrow encryption system from the perspective of supply chain. IEEE Access.

[17] Din I.U., Almogren A., Han Z., Guizani M. (2025). Ensuring privacy and integrity in IoT supply chains through blockchain and homomorphic encryption. IEEE Internet Things J..

[18] Li C., Shen H., Shi X., Liang H. (2023). Quantum secure undeniable signature for blockchain-enabled cold-chain logistics system. Comput. Mater. Contin..

[19] Mouléry M., Sanz Sanz E., Debolini M., Napoléone C., Josselin D., Mabire L., Vicente-Vicente J.L. (2022). Self-sufficiency assessment: Defining the foodshed spatial signature of supply chains for beef in avignon, france. Agriculture.

[20] Zhan Q., Luo M., Qiu M. (2024). An efficient multi-mode certificateless ring signcryption scheme in vanets. IEEE Internet Things J..

[21] Zhou X., Luo M., Qiu M. (2024). A heterogeneous ring signcryption scheme with privacy protection and conditional tracing for smart grid. Comput. Commun..

[22] Wei J., Xie L., Zhu Q., Gao Y., Yu K., Choo K.K.R. (2025). IDTRSC: ID-based traceable ring signcryption framework for data sharing without key escrow. IEEE Trans. Veh. Technol..

[23] Xiong F., Xiao R., Ren W., Zheng R., Jiang J. (2019). A key protection scheme based on secret sharing for blockchain-based construction supply chain system. IEEE Access.

[24] Kalyani D., Srivani P., Pradeep S. (2022). Secured information sharing in supply chain management: Modified data sanitization with optimal key generation via hybrid algorithm. Adv. Eng. Softw..

[25] Vangala A., Das A.K., Mitra A., Das S.K., Park Y. (2022). Blockchain-enabled authenticated key agreement scheme for mobile vehicles-assisted precision agricultural IoT networks. IEEE Trans. Inf. Forensics Secur..

[26] Yang Z., Zolanvari M., Jain R. (2023). A survey of important issues in quantum computing and communications. IEEE Commun. Surv. Tutor..

[27] Shor P.W. (1999). Polynomial-time algorithms for prime factorization and discrete logarithms on a quantum computer. SIAM Rev..

[28] Grover L.K. (1997). Quantum mechanics helps in searching for a needle in a haystack. Phys. Rev. Lett..

[29] Gharavi H., Granjal J., Monteiro E. (2024). Post-quantum blockchain security for the Internet of Things: Survey and research directions. IEEE Commun. Surv. Tutor..

[30] Sendrier N. (2017). Code-based cryptography: State of the art and perspectives. IEEE Secur. Priv..

[31] Butin D. (2017). Hash-based signatures: State of play. IEEE Secur. Priv..

[32] Dey J., Dutta R. (2023). Progress in multivariate cryptography: Systematic review, challenges, and research directions. ACM Comput. Surv..

[33] Nejatollahi H., Dutt N., Ray S., Regazzoni F., Banerjee I., Cammarota R. (2019). Post-quantum lattice-based cryptography implementations: A survey. ACM Comput. Surv. (CSUR).

[34] Zheng Y. (1997). Digital signcryption or how to achieve cost (signature & encryption)≪ cost (signature)+ cost (encryption). Proceedings of the 17th Annual International Cryptology Conference, CRYPTO’97.

[35] Ali R., Obaidat M.S. (2025). Secure and efficient lattice-based signcryption for blockchain-enabled IoT healthcare. IEEE Trans. Dependable Secur. Comput..

[36] Xu S., Chen X., Guo Y., Yiu S.M., Gao S., Xiao B. (2024). Efficient and secure post-quantum certificateless signcryption with linkability for IoMT. IEEE Trans. Inf. Forensics Secur..

[37] Yang X., Cao H., Li W., Xuan H. (2019). Improved lattice-based signcryption in the standard model. IEEE Access.

[38] Yu H., Bai L., Hao M., Wang N. (2020). Certificateless signcryption scheme from lattice. IEEE Syst. J..

[39] Yadav V.K. (2024). Anonymous and linkable ring signcryption scheme for location-based services in vanets. Veh. Commun..

[40] Sourav, Ali R. (2024). Lattice-based ring signcryption scheme for smart healthcare management. Clust. Comput..

[41] Bai Y., He D., Yang Z., Luo M., Peng C. (2025). Efficient module-lattice-based certificateless online/offline signcryption scheme for internet of medical things. IEEE Internet Things J..

[42] Prajapat S., Kumar D., Kumar P., Das A.K., Hossain M.S. (2025). A lattice-based ring signcryption scheme for secure communication in 6G-enabled vehicular ad hoc networks using blockchain. IEEE Trans. Intell. Transp. Syst..

[43] Micciancio D., Regev O. (2009). Lattice-based cryptography. Post-Quantum Cryptography.

[44] Ducas L., Durmus A., Lepoint T., Lyubashevsky V. (2013). Lattice signatures and bimodal Gaussians. Proceedings of the 33rd Annual International Cryptology Conference.

[45] Bert P., Fouque P.A., Roux-Langlois A., Sabt M. (2018). Practical implementation of ring-SIS/LWE based signature and IBE. Proceedings of the 9th International Workshop on Post-Quantum Cryptography, PQCrypto 2018.

[46] Micciancio D., Mol P. (2011). Pseudorandom knapsacks and the sample complexity of LWE search-to-decision reductions. Proceedings of the 31st Annual International Cryptology Conference, CRYPTO 2011.

[47] Micciancio D., Peikert C. (2012). Trapdoors for lattices: Simpler, tighter, faster, smaller. Proceedings of the 31st Annual International Conference on the Theory and Applications of Cryptographic Techniques.

